# Residual cholesterol correlates with mild cognitive impairment, particularly for executive dysfunction among male individuals with type 2 diabetes mellitus

**DOI:** 10.3389/fendo.2026.1890755

**Published:** 2026-08-12

**Authors:** Yafen Chu, Minghui Chen, Qingyue Gao, Ni Lu, Yu Chang, Xiqiao He, Li Ding

**Affiliations:** 1 Department of Endocrinology, Ningbo Medical Center Lihuili Hospital, Ningbo, China; 2 Department of Endocrinology, Wuhan Asia General Hospital, Wuhan, China; 3 Department of Endocrinology and Metabolism, The First Affiliated Hospital of USTC, Division of Life Sciences and Medicine, University of Science and Technology of China, Hefei, China; 4 Department of Endocrinology and Metabolism, Anhui Provincial Hospital Affiliated to Bengbu Medical University, Hefei, China; 5 Department of Endocrinology, The First Affiliated Hospital of Jinzhou Medical University, Jinzhou, China; 6 Department of Geriatrics, The First Affiliated Hospital of Jinzhou Medical University, Jinzhou, China; 7 Department of Endocrinology, Taikang Xianlin Drum Tower Hospital, Affiliated Hospital of Medical School, Nanjing University, Nanjing, China

**Keywords:** lipids metabolism, mild cognitive impairment, nutrition, residual cholesterol, type 2 diabetes mellitus

## Abstract

**Background:**

Residual cholesterol (RC) is derived from lipids, especially for cholesterol metabolism indexes, correlated with excessive nutrient intake, has recently been suggested to influence cognitive function. It not only reflects the nutrition metabolism status, but also is closely associated with diabetic complications. Despite this, how this nutrition-related metabolic marker relates to cognitive dysfunction in type 2 diabetes mellitus (T2DM) patients is still poorly understood. This study sought to assess the link between RC levels and mild cognitive impairment (MCI) among those living with T2DM.

**Methods:**

A total of 195 individuals with T2DM participated in the study and were divided by cognitive status into a normal cognition control group and an MCI group. Between these two cohorts, baseline features were compared. To determine the association between RC and cognitive impairment, both correlation analysis and logistic regression were applied. Sex-based subgroup analyses were conducted, and ROC curve analysis was used to evaluate the diagnostic performance of RC in detecting MCI.

**Results:**

RC levels were notably higher in individuals with MCI than in their cognitively normal counterparts. Even after accounting for age, sex, and how long diabetes had lasted, elevated RC stood out as an independent risk factor for MCI. Among males, RC showed a negative link with MoCA scores—a marker of overall cognitive performance—as well as with verbal fluency test results, pointing to executive deficits. No such relationship emerged in females. According to ROC curve analysis, an RC cutoff of 0.675 mmol/L yielded 80.0% sensitivity and 68.3% specificity (AUC = 0.784) for detecting MCI in the male subgroup.

**Conclusions:**

In male patients with T2DM, elevated RC is one of the risk factors for early cognitive dysfunction, especially affecting the executive function, and a promising biomarker for MCI detection.

## Introduction

Type 2 diabetes mellitus (T2DM) involves not just persistent high blood sugar and reduced insulin sensitivity, but also often coexists with disturbances in nutrient processing, particularly involving lipid metabolism (1). Of these, disrupted cholesterol homeostasis stands out as a key driver—impacting not just the emergence and worsening of diabetes, but also fostering neural complications, such as those involving the nervous system (2, 3). Our earlier work demonstrated that in people with T2DM, high cholesterol correlates with cognitive decline, and raised low-density lipoprotein cholesterol (LDL-C) stands alone as a risk factor for mild cognitive impairment (MCI) (4). Along with LDL-C, decreased concentrations of high-density lipoprotein cholesterol (HDL-C) are similarly related to cognitive decline among diabetic individuals (5). A small sample study shown that total cholesterol (TC) levels are also associated with cognitive function (6). In a separate large-sample analysis, cholesterol levels exhibited a possible U-shaped relationship with cognitive performance among diabetic individuals. Elevated cholesterol levels may impair executive function, while cholesterol deficiency may lead to deficits in visuospatial abilities (7). Cognition may be shaped by circulating cholesterol markers, such as TC, LDL-C, and HDL-C. Both excessively high and abnormally low cholesterol levels may contribute to cognitive impairment. Given the complex relationship between cholesterol metabolism and cognitive function, further research is urgently needed to clarify the link between cholesterol levels and cognitive dysfunction, particularly in patients with T2DM who exhibit disordered lipid metabolism.

The nonfasting lipid fraction carried by triglyceride-enriched lipoproteins—namely, very low-density and intermediate-density particles—is referred to as remnant cholesterol (RC). It reflects the nutrition metabolism status, has recently emerged as a significant contributor to diabetic complications, including atherosclerosis (8) and cardiovascular events (9), stroke (10–12), diabetic nephropathy (13, 14), diabetic retinopathy (15, 16). A multicenter prospective study has shown that RC is closely associated with post-stroke cognitive function (17). Evidence suggests a relationship between diabetes-related cognitive decline and the presence of small-vessel or microvascular disease (18–20). Consistent with prior observations, diabetic microvascular complications such as nephropathy and retinopathy show a robust association with RC concentrations. In line with this, our latest data imply that individuals having both T2DM and diabetic kidney disease face a heightened risk of MCI (21). This potentially indicates that RC play a role in the process of cognitive dysfunction in diabetes. Indeed, a small cross-sectional study performed in China found a correlation between RC and MCI, independent of diabetes status (22). Similarly, a recent study, which was conducted in the United States reported a relation between RC and patients’ verbal learning and memory function (23). Moreover, an analysis based on the NHANES database involving 1,331 participants also suggested a close relationship between RC and cognitive performance (24).

Growing evidence from genetic and epidemiological studies has established RC as a causal risk factor for diabetic complication independent of conventional lipid measures (25). Given the associations between diabetic cognitive impairment and other microvascular complications, the link between lipids (26), including RC and diabetic microvascular complications, and the potential connection between RC and cognitive dysfunction, we hypothesized that RC may be associated with cognitive impairment and could be one of the risk factors for cognitive dysfunction in patients with T2DM. Therefore, in this study, our initial analysis focused on how RC relates to MCI among those living with T2DM. Previous research has shown that both diabetes itself and the development of its complications exhibit sex-related differences (27, 28). Cholesterol metabolism, in particular, is influenced by sex (29), with sex hormones playing a significant role in the regulation of residual cholesterol metabolism (30). Based on this, we conducted a subgroup analysis according to sex. Finally, to further explore the relationship between RC and MCI, we evaluated the diagnostic performance of RC for detecting MCI in male versus female individuals with T2DM, conducting separate analyses by sex.

## Methods

### Experiment design

This present cross-sectional investigation was carried out at the First Affiliated Hospital of USTC. Totally, 198 participants meeting the diagnostic criteria for T2DM were enrolled. Among them, 67 individuals were diagnosed as MCI, while the remaining 128 exhibited no signs of cognitive decline. Based on these assessments, subjects were identified as either MCI group or control group accordingly.

### Ethics

Before initiating the study, all subjects received a comprehensive explanation of the clinical procedures involved. After obtaining written informed consent, all participants confirmed their agreement by signing. The Ethics Committee for Medical Research of the First Affiliated Hospital of USTC examined and authorized the study procedure (No.: 2023-RE-292). Furthermore, this work was conducted according to the Declaration of Helsinki.

### Inclusion and exclusion criteria

All participants enrolled in this investigation met the diagnostic standards for diabetes established under the World Health Organization’s 1999 criteria (31) and had been living with the condition for more than three years. Based on the diagnostic criteria set forth by the Alzheimer’s Disease-focused European Consortium’s MCI Working Group, a total of 67 individuals were identified as having MCI (32). The exclusion parameters adhered to those employed in a prior published study (33).

### Clinical data

A record was made of key participant details—such as age, gender, duration of diabetes mellitus (DMM), hypertension status, height, and weight —along with other demographic and clinical indexes in this present investigation. Body mass index (BMI) was calculated by the formula [BMI = weight (kg)/height (m^2^)]. Levels of glycated hemoglobin (HbA1c) were used as a marker of glucose metabolism. Lipid metabolism was assessed using TG, TC, HDL-c, and LDL-c. Renal function was evaluated based on serum uric acid (UA) and serum creatinine (Cr). RC was determined using the formula (RC = TC − LDL-c − HDL-c). All variables were extracted from electronic medical records.

### Neurocognitive performance

Cognitive function was comprehensively assessed utilizing a suite of validated neuropsychological methods aligned with a published research (34). To assess overall cognition, the Montreal Cognitive Assessment (MoCA) was administered. In line with standard correction protocols, individuals who had completed fewer than twelve years of formal education received an adjusted score. The Trail Making Test–Part A (TMTA) was used to measure information processing speed. Executive function was assessed using the Digit Span Test (DST), the Verbal Fluency Test (VFT), and the Trail Making Test–Part B (TMTB). Immediate and delayed recall were evaluated through the Immediate Recall (AVLT-IR) and Delayed Recall (AVLT-DR) components, respectively. Emotional memory was assessed with the Logical Memory Test (LMT).

### Statistical methods

All statistical work was performed using SPSS (version 22.0; IBM Corp., USA). Data following a normal distribution are shown as mean ± SD. Between-group comparisons for such variables (patients with vs. without MCI) were made using Student’s t-test. Variables deviating from normality are presented as median and interquartile range, with group differences examined via the Mann–Whitney U test. Categorical data are reported as counts and percentages, compared using the chi-square test. Pearson or partial correlation analysis was used to explore links between RC and specific cognitive measures. Binary logistic regression identified possible independent predictors of MCI. Receiver operating characteristic (ROC) curves assessed diagnostic performance (sensitivity, specificity, and optimal cutoff) for MCI.

## Results

### Clinical data of T2DM patients with and without MCI

Although this is a cross-sectional study without strict matching for age, sex, or DMM, the two groups remained comparable in age, sex composition, and DDM (all P > 0.05). Likewise, no meaningful differences emerged in BMI or the proportion of hypertension (all P > 0.05). Although patients with MCI tended to exhibit slightly elevated HbA1c and serum UA levels, these differences did not reach statistical significance (P = 0.095 and P = 0.063, respectively). Likewise, serum Cr concentrations did not differ meaningfully between cognitively impaired and cognitively intact individuals. We then focused on evaluating disparities in lipid profiles. While TC, TG), and LDL-c levels were comparable between groups (all P > 0.05), individuals with MCI demonstrated significantly reduced HDL-c and increased RC concentrations (P = 0.001 and P = 0.009, respectively) (**Table 1**).

**Table 1:** Comparation of clinical parameters and neurophysiological test results between Control and MCI groups.

	Control group (n = 128)	MCI group (n = 67)	P
Age (year)	63.00 (55.25, 71.00)	61.00 (55.00, 69.00)	0.581 ^a^
Female (n, %)	68, 53.13%	32, 47.76%	0.477 ^b^
DDM (year)	10.00 (6.00, 18.00)	10.00 (8.00, 18.00)	0.542 ^a^
Hypertension (n, %)	90, 70.31%	53, 79.10%	0.187 ^b^
BMI (kg/m^2^)	24.30 (21.94, 26.16)	24.74 (22.66, 27.34)	0.512 ^a^
HbA1c (%)	7.60 (6.90, 8.68)	8.40 (6.90, 9.70)	0.094 ^a^
SUA	301.10 (255.15, 344.93)	316.60 (281.00, 365.00)	0.063 ^a^
Cr	63.00 (53.25, 72.00)	65.00 (54.00, 78.00)	0.334 ^a^
TG	1.40 (0.94, 1.97)	1.68 (1.13, 2.49)	0.119 ^a^
TC	4.47 ± 1.03	4.44 ± 0.83	0.862 ^c^
LDL-c	2.68 ± 0.79	2.60 ± 0.70	0.520 ^c^
HDL-c	1.13 (0.98, 1.30)	1.01 (0.85, 1.14)	0.001 ^a*^
RC	0.63 ± 0.32	0.84 ± 0.60	0.009 ^c*^
MoCA	28.00 (27.00, 29.00)	25.00 (24.00, 25.00)	< 0.001 ^a*^
DST	12.00 (10.00, 15.00)	11.00 (9.00, 12.00)	< 0.001 ^a*^
VFT	17.54 ± 3.98	13.16 ± 4.53	< 0.001 ^c*^
CDT	3.00 (2.00, 4.00)	2.00 (2.00, 3.00)	< 0.001 ^a*^
TMTA	59.00 (49.00, 74.75)	73.00 (57.00, 84.00)	< 0.001 ^a*^
TMTB	158.38 ± 51.69	185.37 ± 51.78	< 0.001 ^c*^
AVLT-IR	17.00 (24.00, 21.00)	15.00 (12.00, 18.00)	0.041 ^a*^
AVLT-LR	8.00 (6.00, 12.00)	6.00 (5.00, 7.00)	< 0.001 ^a*^
LMT	8.50 (6.00, 12.00)	7.00 (4.00, 9.00)	0.032 ^a*^

^a^ The Mann-Whitney U test was employed for asymmetrically distributed variables; ^b^ The Chi-square test was employed for categorical variables; ^c^ Student’s t test was employed for normally distributed variables. *P<0.05. MCI, mild cognitive impairment; DDM, duration of diabetes mellitus; BMI, body mass index; HbA1c, glycosylated hemoglobin; SUA, serum uric acid; Cr, creatinine; TG, triglycerides; TC, total cholesterol; LDL-c, low density lipoprotein cholesterol; HDL-c, high density lipoprotein cholesterol; RC, residual cholesterol; MoCA, Montreal cognitive assessment; DST, digit span test; VFT, verbal fluency test; CDT, clock drawing test; TMTA, trail making test-A; TMTB, trail making test-B; AVLT-IR, auditory verbal learning test-immediate recall; AVLT-DR, auditory verbal learning test-delayed recall; LMT, logical memory test.

### Difference of neurocognitive examination results in T2DM patients with and without MCI

To confirm the presence of MCI in individuals with T2DM, a battery of Neurocognitive evaluations was administered. Patients with co-occurring MCI showed a marked drop in their MoCA results, pointing to an overall deterioration in cognitive function. Furthermore, TMTA completion times were markedly prolonged, reflecting slower information processing speed. Measures of executive function—including the DST and VFT—demonstrated significant impairments, while the TMTB, another indicator of executive functioning, also showed a pronounced increase in completion time. Additionally, scores on the AVLT-IR and AVLT-DR, which assess memory function, were significantly reduced. The LMT scores, reflecting narrative memory performance, similarly showed a noticeable decline (all *P* < 0.05) (**Table 1**).

### Association between RC and cognitive function in all patients with T2DM

To explore how RC relates to cognitive performance among people with T2DM, a preliminary Pearson correlation was performed on the entire populations. The findings revealed an inverse relationship between RC levels and global cognitive ability, as assessed by the MoCA scores (R = −0.143, P = 0.046). Modest negative trends were also observed between RC and specific cognitive measures such as the DST (R = −0.131, P = 0.069), VFT (R = −0.131, P = 0.067), and AVLT-LR (R = −0.123, P = 0.086) scores, though these associations did not reach statistical significance. In contrast, no meaningful correlation was detected between RC and other cognitive indices, including the CDT, TMTA, TMTB, AVLT-IR, and LMT scores (all P > 0.05). Although no significant differences were found between T2DM patients with and without MCI in terms of age, sex, or DDM, the cross-sectional nature of the study warranted further investigation using partial correlation analysis. After adjusting for age, sex, and DDM, a stronger negative correlation emerged between RC and MoCA scores (R = −0.168, P = 0.020), suggesting a more robust link between elevated RC and diminished global cognitive function (**Table 2**).

**Table 2:** Association between RC and cognitive performance in patients with T2DM.

	Model 1		Model 2	
	R	P	R	P
MoCA	-0.143	0.046^*^	-0.168	0.020^*^
DST	-0.131	0.069	-0.136	0.060
VFT	-0.131	0.067	-0.140	0.054
CDT	-0.015	0.832	-0.044	0.541
TMTA	0.036	0.620	0.042	0.559
TMTB	0.018	0.808	0.007	0.919
AVLT-IR	0.055	0.448	0.041	0.571
AVLT-LR	-0.123	0.086	-0.121	0.096
LMT	0.070	0.333	0.056	0.442

^*^ P<0.05. Model 1 Pearson association between RC and cognitive performance in patients with T2DM: Model 2: Partial association between RC and cognitive performance in patients with T2DM adjusting for age, gender, and DDM.

### Association between RC and cognitive function in female and male patients with T2DM

As noted in the introduction, sex-based differences may play a role in diabetic cognitive impairment, and sex hormones are closely intertwined with cholesterol metabolism. To further investigate this, we conducted sex-stratified analyses to examine the association between remnant cholesterol levels and cognitive performance scores. In men, RC was negatively linked to both the MoCA (R = −0.334, P < 0.001; R = −0.318, P = 0.002, respectively)—a measure of overall cognitive performance—and the VFT (R = −0.238, P = 0.020; R = −0.209, P = 0.045, respectively), an indicator of executive function. These relationships persisted even after controlling for age and how long diabetes had lasted. By contrast, no such associations appeared among women (**Table 3**).

**Table 3:** Association between RC and cognitive performance in female and male patients with T2DM.

	Female				Male			
	Model 1		Model 2		Model 1		Model 2	
	R	P	R	P	R	P	R	P
MoCA	0.126	0.211	0.086	0.402	-0.334	< 0.001^*^	-0.318	0.002^*^
DST	-0.074	0.467	-0.101	0.322	-0.174	0.092	-0.147	0.159
VFT	0.024	0.812	-0.008	0.938	-0.238	0.020^*^	-0.209	0.045^*^
CDT	0.028	0.779	-0.003	0.973	-0.039	0.705	-0.059	0.576
TMTA	-0.034	0.738	-0.025	0.806	0.081	0.435	0.083	0.427
TMTB	-0.004	0.971	-0.010	0.918	0.035	0.737	0.013	0.899
AVLT-IR	0.091	0.368	0.071	0.486	0.029	0.780	0.044	0.675
AVLT-LR	-0.058	0.563	-0.066	0.521	-0.173	0.094	-0.156	0.135
LMT	0.129	0.202	0.116	0.254	0.050	0.633	0.029	0.780

^*^ P<0.05. Model 1 Pearson association between RC and cognitive performance in female and male patients with T2DM: Model 2: Partial association between RC and cognitive performance in female and male patients with T2DM adjusting for age, gender, and DDM.

### Exploring the risk factor of MCI in patients with T2DM

To investigate whether RC serves as a potential risk factor for MCI in individuals with T2DM, binary logistic regression analyses were conducted separately in the total cohort, as well as stratified by sex. In the overall population, elevated levels of RC were consistently associated with increased odds of MCI, regardless of adjustment for age, sex, and DDM, indicating an independent relationship (P = 0.006, OR = 3.343; P = 0.006, OR = 3.592, respectively) (**Table 4**). Subgroup analysis further revealed that this association remained significant among male patients with T2DM, even after controlling for age and DDM (P < 0.001, OR = 78.710; P < 0.001, OR = 64.853, respectively). In contrast, no statistically significant association was observed between RC levels and MCI in female participants, irrespective of covariate adjustment. These findings suggest a potential sex-specific role of remnant cholesterol in the cognitive decline observed in T2DM (**Table 5**).

**Table 4:** Assessment of risk factors for MCI by binary logistic analysis in all patients with T2DM.

		β	SE	P	OR	95% CI	
						Lower	Upper
Model 1	RC	1.207	0.493	0.006^*^	3.343	1.415	7.896
Model 2	RC	1.279	0.416	0.006^*^	3.592	1.454	8.870
	Age	0.010	0.019	0.584	1.010	0.974	1.048
	Gender	0.208	0.315	0.508	1.231	0.664	2.283
	DDM	0.005	0.022	0.815	1.005	0.963	1.049

^*^ P<0.05. Model 1 Assessment of risk factors for MCI by binary logistic analysis in all patients with T2DM: Model 2: Assessment of risk factors for MCI by binary logistic analysis in all patients with T2DM adjusting for age, gender, and DDM.

**Table 5:** Assessment of risk factors for MCI by binary logistic analysis in female and male patients with T2DM.

			β	SE	P	OR	95% CI	
							Lower	Upper
Female	Model 1	RC	-0.608	0.702	0.386	0.544	0.138	2.153
	Model 2	RC	-0.404	0.730	0.580	0.668	0.160	2.792
		Age	0.046	0.026	0.081	1.047	0.994	1.102
		DDM	-0.003	0.031	0.912	0.997	0.938	1.059
Male	Model 1	RC	4.366	1.182	< 0.001^*^	78.710	7.755	798.832
	Model 2	RC	4.172	1.217	< 0.001^*^	64.853	5.971	704.345
		Age	-0.018	0.031	0.549	0.982	0.925	1.043
		DDM	0.017	0.034	0.620	1.017	0.951	1.088

^*^ P<0.05. Model 1: Assessment of risk factors for MCI by binary logistic analysis in female and male patients with T2DM; Model 2: Assessment of risk factors for MCI by binary logistic analysis in female and male patients with T2DM adjusting for age, gender, and DDM.

### Evaluation of the diagnostic value of RC for MCI in patients with T2DM

**Figure 1:**
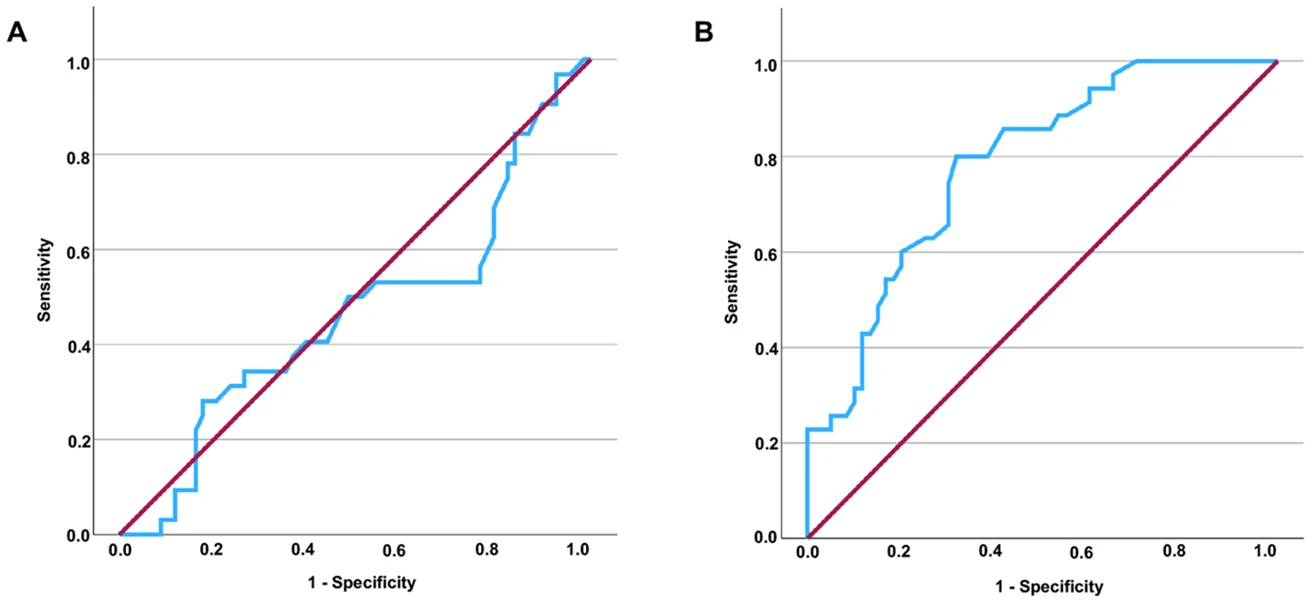
ROC curve of RC for the sensitivity and specificity of MCI in female and male patients with T2DM. The area under the curve is 0.656 in female patients with T2DM **(A)**. Additionally, the area under the curve is 0.784 in male patients with T2DM **(B)**. It is determined that the diagnostic cut-off value for RC to be 0.675 mmol/l, revealed a sensitivity of 80.0% and specificity of 68.3% in male patients with T2DM.

Based on our findings that elevated RC increases the likelihood of MCI in men who have T2DM, we further explored the potential diagnostic utility of RC by ROC. The analysis revealed a notably limited diagnostic value of RC among female T2DM patients, yielding an area under the curve (AUC) of only 0.466 (**Figure 1**). However, we the cut-off point of 0.675 mmol/l achieving a specificity of 68.3%, a sensitivity of 80.0%, and an AUC of 0.784 for MCI in male patients with T2DM (**Figure 1**).

## Discussion

In this cross-sectional study, we explored the link between RC levels and cognitive dysfunction in patients with T2DM. Our main findings demonstrated that patients with T2DM and MCI exhibited significantly lower HDL-c and higher RC levels compared to patients without cognitive function. Moreover, RC was inversely associated with global cognition, particularly in male patients, and independently increased the risk of MCI in this subgroup. Although no significant differences were observed in demographic variables, DMM, or conventional metabolic risk factors such as BMI, hypertension, or glycemic control between groups, patients with MCI and other diabetic complications tended to have higher HbA1c and uric acid levels, which is consistent with our previous studies (35–39), highlighting the contribution of chronic hyperglycemia and metabolic dysregulation to cognitive decline in T2DM. However, these associations did not reach statistical significance in our research, suggesting that additional lipid-related mechanisms may underlie cognitive vulnerability in this population.

RC, a cholesterol fraction carried primarily by triglyceride-rich lipoproteins such as VLDL and IDL, is increasingly recognized as a pro-atherogenic lipid component (40). Elevated RC contributes to endothelial dysfunction (41), low-grade inflammation (42), and impaired microvascular circulation (43), all of which may exacerbate neurovascular injury and accelerate cognitive decline in T2DM. Our analysis highlighted RC as a potential lipid marker linked to cognitive impairment. Our investigation demonstrated that patients with T2DM who also exhibited MCI had elevated levels of RC. Correlation analysis further revealed an inverse relationship between RC concentrations and MoCA performance, which serves as a measure of global cognitive ability. Notably, this association persisted after adjustment for potential confounders, suggesting that RC may act as an independent contributor to cognitive decline and a possible mechanistic factor, rather than simply reflecting lipid abnormalities. Consistent with this, our prior work has indicated that other lipid-related markers (7–44) and genetic variants (4) might also be implicated in cognitive function.

In the present analysis, RC exhibited only a marginal correlation with overall cognitive outcomes, as assessed by MoCA scores across the entire population. Furthermore, prior work has drawn attention to marked distinctions between sexes regarding how diabetes and its related complications arise and progress biologically (27, 28). Evidence indicates that sex hormones play a role in regulating lipid metabolism (45, 46). Moreover, additional studies suggest that hormonal influences may also extend to cognitive processes (47, 48). These results implying that sex-specific factors could underlie variations in the relationship between cognitive function and RC. This finding led us to explore if dividing by sex could uncover more pronounced links in a particular subgroup. In line with this, separate analyses by sex subsequently showed that the association between RC and cognitive decline reached significance exclusively among male patients. Elevated RC independently predicted MCI risk among men, with a remarkably high odds ratio, while no association was observed in women. This sex-specific difference may be partly explained by the modulatory effects of sex hormones on lipid metabolism and cognitive resilience.

From a clinical perspective, our ROC analysis suggests that RC may have moderate diagnostic value for identifying MCI in male T2DM patients, with an AUC of 0.784 and acceptable sensitivity and specificity. In contrast, RC showed poor discriminative ability among women, reinforcing the need for sex-specific risk stratification. Although RC alone may not serve as a universal biomarker, its integration into multi-marker panels could enhance the early detection of cognitive impairment in at-risk male populations with T2DM.

There are several limitations should be recognized. Firstly, the sample size is fairly small, which could reduce statistical power when identifying weaker relationships, especially within subgroup evaluations. As a cross-sectional study, this work is inherently limited in its research value relative to case–control studies, owing to the absence of strict age and sex matching. While no significant age difference was observed between the two groups, and age was adjusted for in subsequent analyses—with sex-stratified subgroup analyses performed per our study aims—we recognize this as a limitation. Furthermore, unlike randomized controlled trials, our study can only suggest an association between RC and cognitive function, not a causal relationship. Secondly, one limitation of the present study is that we were unable to evaluate the influence of pharmacological treatment. Medication use was considered during the study planning phase, and information on commonly prescribed therapies was collected, including glucose-lowering medications (e.g., metformin and insulin) as well as lipid-lowering agents such as statins. However, the data collection was limited to whether participants were receiving each medication category, without detailed records of treatment dose, duration, or adherence. In addition, the modest sample size and the low frequency of use for several medications restricted our ability to perform meaningful analyses of their individual effects. Consequently, the potential contribution of medication exposure could not be adequately examined and should be acknowledged as a limitation. Notably, a recent network meta-analysis has demonstrated that several antidiabetic therapies may confer cognitive benefits, further supporting the possibility that pharmacological interventions play a role in preserving cognitive function (49). Thirdly, unmeasured factors such as dietary habits, genetic predispositions, and inflammatory markers may confound the observed associations. Fourthly, the retrospective design of this study prevented us from obtaining information on participants’ levels of physical activity. Finally, the neuropsychological battery, while comprehensive, may not capture subtle domain-specific deficits that could be further elucidated with advanced neuroimaging or biomarker studies.

Our previous clinical investigations (4, 7, 44–50) and others (51, 52) have highlighted a strong link between lipid metabolism and cognitive dysfunction, while our experimental studies have further indicated that cholesterol homeostasis plays an important role in cognitive processes (53). RC, representing a lipid fraction beyond conventional parameters, has recently emerged as a novel biomarker for predicting metabolic disturbances and diabetes-related complications. Evidence has suggested an association between RC and various diabetic complications (15–54) and cognitive decline (24). In the present study, we not only confirmed this relationship in individuals with T2DM but also extended the analysis by conducting subgroup comparisons. Our findings revealed that the relationship between RC levels and MCI was restricted to male patients with T2DM, whereas no significant relationship was observed among females. Moreover, we evaluated the diagnostic potential of RC for identifying MCI specifically in the male subgroup.

## Conclusion

In summary, our study provides novel evidence that elevated RC is independently associated with cognitive dysfunction in T2DM, particularly among male patients. These findings highlight the potential utility of RC as a sex-specific biomarker for early identification of cognitive risk and emphasize the importance of integrating lipid management into strategies for preserving cognitive health in diabetic populations.

## Data Availability

The original contributions presented in the study are included in the article/supplementary material. Further inquiries can be directed to the corresponding authors.
